# Release of Biopolymers from *Saccharomyces cerevisiae* Biomass Through Thermal and Non-Thermal Technologies

**DOI:** 10.3390/microorganisms12122596

**Published:** 2024-12-15

**Authors:** Marianna Ciccone, Muhammad Rehan Khan, Junior Bernardo Molina Hernandez, Joel Armando Njieukam, Lorenzo Siroli, Davide Gottardi, Rosalba Lanciotti, Pietro Rocculi, Francesca Patrignani

**Affiliations:** 1 Department of Agricultural and Food Sciences, Campus of Food Science, Alma Mater Studiorum, University of Bologna, Piazza Goidanich 60, 47521 Cesena, Italy; muhammadrehan.khan2@unibo.it (M.R.K.); junior.molina@unibo.it (J.B.M.H.); joelarmando.njieuka2@unibo.it (J.A.N.); lorenzo.siroli2@unibo.it (L.S.); davide.gottardi2@unibo.it (D.G.); rosalba.lanciotti@unibo.it (R.L.); pietro.rocculi3@unibo.it (P.R.); francesca.patrignani@unibo.it (F.P.); 2 Interdepartmental Centre for Agri-Food Industrial Research, Campus of Food Science, Alma Mater Studiorum, University of Bologna, Via Quinto Bucci 336, 47521 Cesena, Italy

**Keywords:** high-pressure homogenisation, pulsed electric field, yeast cell wall disruption, β-glucans extraction, mannoproteins recovery, biopolymers

## Abstract

Components of yeast cell walls, such as β-glucans and mannoproteins, show promise for developing sustainable biopolymers for food packaging. Efficient extraction, however, is challenging due to the complexity of the yeast cell wall. This study explored high-pressure homogenisation (HPH) and pulsed electric fields (PEFs), alone and with heat treatment (TT), on bakery yeast (BY) and brewery spent yeast (BSY) biomasses. In the treated samples we assessed carbohydrates, proteins, β-glucans, and mannoproteins and evaluated cell wall disruption microscopically. HPH caused complete cell disintegration, enhancing intracellular release, while PEF primarily permeabilised the membranes. Combined HPH and PEF treatments significantly increased cell wall stress, leading to partial disintegration. Notably, the β-glucans released reached 3.90 g/100 g dry matter in BY and 10.44 g/100 g dry matter in BSY, demonstrating significant extraction improvements. These findings highlight the potential of HPH and PEF for enhancing β-glucan recovery from yeast biomass, offering a promising route for sustainable biopolymer production for food packaging.

## 1. Introduction

The urgent need to switch towards sustainable polymers to develop food packaging, as opposed to packaging derived from petrochemical origin, has stimulated researchers towards bio-based polymers as an alternative [1]. Technological advancements have made biopolymers increasingly preferred also for extending food shelf life and meeting eco-friendly consumer demands. Agro-industrial sources provide biopolymers like proteins and polysaccharides [2,3], while microbial-origin biopolymers have been identified as a potential source for developing sustainable packaging materials [4,5,6,7,8]. In particular, yeast biomass contains about half of its dry weight in the form of proteins and polysaccharides that could be isolated and used in the formulation of food films [4,9,10]. However, the extensive application of yeast biomass in the creation of films is still mainly unknown. The cell wall of yeast, which accounts for 15–30% of its dry weight, contains important substances such as mannoproteins and β-glucans. Mannoproteins serve as structural elements and account for 35–40% (*w*/*w*) of the dry weight of yeast cell walls [11], and they have emulsifying and stabilizing abilities demonstrated in numerous studies owing to their amphipathic nature. 

Polymers extracted from yeast, such as β-glucans and mannoproteins, offer significant advantages due to their natural bioactivity, including immune-boosting and antioxidant properties. Additionally, they are biodegradable and derived from renewable sources, making them environmentally friendly alternatives for various industrial applications. On the other hand, β-glucans display useful technological properties for the food industry as a thickener and an emulsion stabiliser due to their water-holding capacity, and they make up the majority of the cell wall in yeast (50–55% *w*/*w*) [12,13]. The mechanical properties of these polysaccharides are given by their branched structure (β-(1,3) and β-(1,6)-D-glucan) that provides mechanical stability and film-forming abilities comparable to synthetic materials [14]. The triple-helix structure of yeast-derived β-glucans is unique amongst polysaccharides, offering high tensile strength and effective oxygen barrier properties, essential for safe and effective packaging [4]. 

Although β-glucan isolation can be complex and costly, recent research has demonstrated the successful use of β-glucan concentrates isolated from the cell walls of *Saccharomyces cerevisiae*, which can enhance material yield and provide useful functional properties [4,15]. When combined with other biopolymers, β-glucans can form interconnected networks that enhance the thermal and mechanical properties of films [16], broadening their potential for biodegradable food packaging applications. The scientific interest in β-glucans for packaging is thus motivated by their biodegradability, biocompatibility, and potential antimicrobial and antioxidant properties, positioning them as strong candidates to replace synthetic materials and reduce environmental impact [4,12,17]. This constituent has been the focus of several research papers dealing with the valorisation of spent yeast from brewing and winemaking processes [18,19]. Physical, chemical, and enzymatic approaches to mannoprotein extraction from wasted yeast (*S. cerevisiae*) have been compared and examined by [20]. However, some limitations, such as the scalability of the extraction methods and the cost-effectiveness of producing high-purity biopolymers, are underlined. As a consequence, the valorisation of by-products and the overall reduction in waste generation requires the development of effective, cost-efficient processes. Glucans, mannoproteins, and chitins, which give the cell its mechanical strength, have been identified as the basic structural elements of the yeast cell wall [20,21]. The breakdown of the structural elements of the wall, in this case, the glucans and mannoproteins in yeast, is necessary for the complete dissolution of the cell wall and the release of the internal components. 

Various cell disruption methods have been developed that allow rapid and inexpensive release of these products from their hosts. According to [22], high-pressure homogenisation (HPH) is a mechanical cell disruption process that has a long history of use as a pretreatment for the extraction of valuable intracellular compounds [23,24]. Cell suspensions subjected to HPH are disrupted by turbulence and shear phenomena while being forced through the homogeniser valve [25]. Pressures higher than 60 MPa are generally required for a massive release of cytoplasmatic content [26]. Thereafter, polymers are completely free to interact and form a stable network. Also, pulsed electric fields (PEFs) and Ultrasonication have been employed [27,28,29] for the extraction of intracellular proteins from yeast cells. Protein extraction increased with increasing treatment intensity, and cell debris shifted to smaller particle sizes. PEF processing causes electroporation of cells in aqueous suspensions, resulting in the partial loss of cytoplasmic content. The application of PEF for the extraction of intracellular components such as proteins, enzymes, and polysaccharides from yeast has been studied [25,30]. Electroporation leads to the release of ionic compounds and certain macromolecules without critically affecting the cell morphology of microbial cells [31]. This can both improve extract separability and allow for the valorisation of the insoluble cell wall material for polysaccharide extraction [32]. Despite several authors having reported yeast cell wall disruption to release value-added products through single treatments of HPH [33], PEF [34,35], or thermal treatment (TT), this study represents the first attempt, to the best of our knowledge, to evaluate the release of β-glucans from yeast biomass using a combination of PEF, HPH, and TT. While TT alone is known for its effectiveness in cell wall disruption, it is also highly energy-intensive, making it less favourable in terms of sustainability and cost-effectiveness. By combining PEF and HPH with TT, we aimed to reduce the energy required for effective cell wall disruption while maximising the yield of valuable compounds. The sequential application of these methods allows for improved selectivity in the extraction process and a reduction in the energy demand associated with thermal treatments alone. This synergistic approach enhances the overall process efficiency and cost-effectiveness, as also shown in the case of microalgae processing by [36,37].

In this context, this study explored the use of high-pressure homogenisation (HPH) and pulsed electric field (PEF) treatments, combined or not with traditional thermal treatments (TTs), to disrupt yeast cell walls, aiming to release and recover the highest β-glucan and mannoprotein fractions from the yeast biomass to be further used as innovative biopolymers for food packaging applications. 

## 2. Materials and Methods

### 2.1. Yeast Biomasses

Samples of commercial baker’s yeast (BY) and brewer’s spent yeast (BSY) (*Saccharomyces cerevisiae*) were used in this study. Commercial BY, sourced from the brand *Lievital*, was purchased from a local supermarket (Cesena, Italy), while BSY biomass was provided by a local brewery industry located in Cesena (Italy).

### 2.2. Biomass Treatments 

BY and BSY of *Saccharomyces cerevisiae* (100 g) were dispersed in 1 L of distilled water, resulting in a stock dispersion of 10% *w*/*v* of dry matter, according to [38]. Subsequently, an aliquot of the prepared yeast cells was subjected to various treatments, including high-pressure homogenisation (HPH) (100 mL), pulsed electric field (PEF) (50 mL), and heat treatment (TT) of 90 °C for 20 min (100 mL) using a laboratory autoclave (Vapor Matic mod. 770/A). 

#### 2.2.1. High-Pressure Homogenisation (HPH) Treatment 

The samples were subjected to a high-pressure homogenisation treatment with a continuous high-pressure homogeniser, PANDA (Gea, Parma, Italy). The machine was supplied with a homogenizing PS-type valve; the valve assembly included a ball-type impact head made of ceramics, a stainless steel large inner diameter impact ring, and a tungsten carbide passage head. The inlet temperature of the cell suspensions was 25 °C. The samples were subjected to HPH treatments at 125 MPa for 3 passes, with a tube-in-tube heat exchanger, to minimise the thermal increase in temperature, which was 1.5 °C/10 MPa. This specific treatment protocol was chosen based on the established literature [34]. 

#### 2.2.2. Pulsed Electric Field (PEF) Treatment 

PEF treatment was performed using a lab scale PEF unit Mod. EPULSUS^®^-BM1A-15 delivering a maximum output current and voltage of ±15 kV and 200 A, respectively (Energy Pulse System, Lisboa, Portugal). Fifty millilitres of BY and BSY of *S. cerevisiae* dispersions (conductivity 2 mS/cm) were placed in a rectangular treatment chamber (10 cm length × 10 cm width × 10 cm height) consisting of two parallel stainless steel electrodes (3 mm thick) spaced at 10 cm. The PEF treatments were applied to the samples at 25 °C and were carried out with a monopolar square by applying a series of pulses (45 pulses and 145 µs duration of each pulse) with a fixed field strength of 1.5 kV/cm, pulse width 3 ± 0.9 µs, and frequency 7.5 Hz. The temperature changes due to the PEF treatments were negligible. Finally, the specific energy input was 0.012 ± 0.12 kJ/kg. 

Following the various treatments applied, a series of BY and BSY samples was obtained, as shown in Table 1. The control sample (NT) was represented by the non-treated dispersion. An aliquot of all the dispersions obtained as a result of the different treatments was centrifuged at 19,800× *g* for 15 min (rotor JA-14, Beckman Centrifuge J2-MC, Beckman Coulter Inc., Brea CA, USA), and the obtained supernatant was collected. For each yeast biomass and treatment applied, both dispersions and supernatants were collected and stored at −20 °C until use for further analysis [38].

### 2.3. Microscopic Observation

The dispersions obtained after the different treatments were observed by an Eclipse Ti-U microscope (Nikon Co., Tokyo, Japan) at 100× and 40× magnification. Photos of the preparations were obtained using a digital camera (DS-Qi1Mc, Nikon Co., Tokyo, Japan) and NIS Elements software (Nikon Co., Tokyo, Japan). 

### 2.4. Evaluation of the Material Composition After Different Treatments 

Dry matter, carbohydrates, proteins, β-glucans, and mannoproteins concentration analyses were performed on the dispersions obtained following the different treatments, and on the supernatants obtained from centrifuging the treated and untreated dispersions.

#### 2.4.1. Dry Matter 

The dry matter content was determined gravimetrically for all samples, both on the dispersions and the supernatants obtained after centrifugation. The supernatants and dispersions were dried at 105 °C and weighed in an analytical balance ±0.001 to constant weight. Analyses were conducted in triplicate after three biological repetitions [38].

#### 2.4.2. Carbohydrates (CH) Concentration 

The carbohydrate content (CH) was determined using the Dubois method [39]. This method involves the dehydration of carbohydrates in an acidic environment, producing furan derivatives that react with phenol to form coloured compounds. A 1 mL sample was mixed with a 5% *w*/*v* phenol solution (0.5 mL) and homogenised by the vortex. Subsequently, 2.5 mL of concentrated H_2_SO_4_ was added and homogenised again. The mixture was incubated for 10 min at room temperature and then for 15 min at 37 °C. After cooling, the absorbance of the appropriately diluted samples was measured at 490 nm. A calibration curve was prepared using glucose solution as a standard in the range of 0 to 80 μg/mL. 

#### 2.4.3. Proteins Concentration 

The protein concentration was measured using the Bradford Protein Analysis Kit (Bio-Rad; Hercules, CA, USA). This spectrophotometric analysis is based on the binding of the Coomassie Brilliant Blue dye present in the Bradford solution with the basic residues of amino acids such as arginine, lysine, and histidine, causing a dark red to blue colour change. The protein concentration calculation was based on a standard curve using bovine serum albumin (BSA), and the concentration of the samples was expressed in mg/mL [40,41].

#### 2.4.4. β-(1,3)(1,6)-glucan Content 

The β-(1,3)(1,6)-glucan content of the dispersions and supernatants was determined using an Enzymatic Yeast β-Glucan Kit (K-EBHLG, Megazyme, Bray, Ireland) according to the method proposed by [42]. Prior to analysis, the liquid supernatants were dried in an oven at a maximum temperature of 60 °C to prevent any degradation of the β-glucans [43]. Approximately 20 mg of treated samples were then solubilised and hydrated in 2 N KOH during incubation on a magnetic stirrer in an ice-water bath. After 30 min, the solution was subsequently adjusted to pH 4.0–4.5 with 1.2 M sodium acetate buffer. Then, the slurry was incubated for 16 h at 40 °C with Glucazyme enzyme mixture (exo-1,3-β-glucanase, endo-1,3-β-glucanase, β-glucosidase, and chitinase). After dilution and centrifugation, an aliquot was removed for determination of glucose with the GOPOD reagent, consisting of glucose oxidase, peroxidase, and 4-amino-antipyrine in buffer prepared using p-hydroxybenzoic acid and sodium azide (0.4% *w*/*v*, pH 7.4). The free glucose was oxidised by glucose oxidase to gluconic acid and to hydrogen peroxide. Next, the hydrogen peroxide was reduced by peroxidase while the 4-aminoantipyrine was oxidised to a coloured product, of which the absorbance was measured. The absorbance of samples and a standard of D-glucose (1.5 mg/cm^3^) were measured at 510 nm against a reagent blank. Calculations of β-glucan content were based on Formula (1):

(1) $$\beta \left(1,3\right)\left(1,6\right)-glucan\;\left(\%\frac{w}{w}\right)=∆E\times F\times \frac{12.04}{0.1}\times \frac{100}{W}\times \frac{1}{1000}\times \frac{162}{180}$$

where ΔE is the absorbance measured against the blank, F is the conversion of absorbance to µg (150 µg D-glucose) standard divided by the GOPOD absorbance of this 150 µg, 12.04/0.1 is the volume correction (0.1 mL from 12.04 mL), 1/1000 is the conversion from µg to mg, W is the weight of the analysed sample in mg, 100/W is the factor to represent β-(1,3)(1,6)-glucan as a percentage of the sample weight, and 162/180 is the factor to convert free D-glucose to anhydro-D-glucose as found in β-(1,3)(1,6)-glucan.

#### 2.4.5. Mannoproteins Content 

Mannoproteins are composed of mannose units linked to polypeptide chains. The release of mannoproteins was indirectly assessed by measuring the concentration of mannose in the dispersions and supernatant [35,44,45]. This was achieved through hydrolysis with sulphuric acid (resulting in a final concentration of 1.5 M) at 100 °C for 90 min, followed by neutralisation with NaOH (2 M). During this process, the mannose chains that make up the mannoproteins are broken down into their monomeric form. The quantitative analysis of mannose concentration was performed using an enzymatic method by utilizing a spectrophotometer at a wavelength of 340 nm, specifically through the D-Mannose, D-Fructose, D-Glucose Assay kit (Megazyme International, Wicklow, Ireland).

### 2.5. Determination of the Dispersible Index (DI%)

From the carbohydrate, protein, β-glucan, and mannoprotein concentration values measured in both the dispersions and supernatants, it was possible to determine the dispersible index (DI%). This index represents the percentage of each component originally present in the native yeast that was released into the supernatant after treatment, calculated using Equation (2):

(2) $$DI\left(\%\right)=\frac{\%component\;in\;supernatant\;}{\%component\;in\;initial\;yeast\;suspension\;}\times 100$$

The numerator and denominator refer to the percentage of a given compound measured in equivalent volumes of suspension. A DI% of 100% would indicate the complete transfer of the component from the cells to the supernatant, providing a clear metric for the release efficiency [38].

### 2.6. Statistical Analysis

The data were analysed using the one-way analysis of variance (ANOVA) of the Statistica software (version 8.0; Statsoft, Tulsa, OK, USA). Subsequently, the mean values of dry matter, carbohydrates, proteins, β-glucans, and mannoproteins of the different samples were differentiated using the Tukey honest significant difference (HSD) test, considering a significance level of (*p* < 0.05).

## 3. Results and Discussion

### 3.1. Microscopic Observations

**Figure 1:**
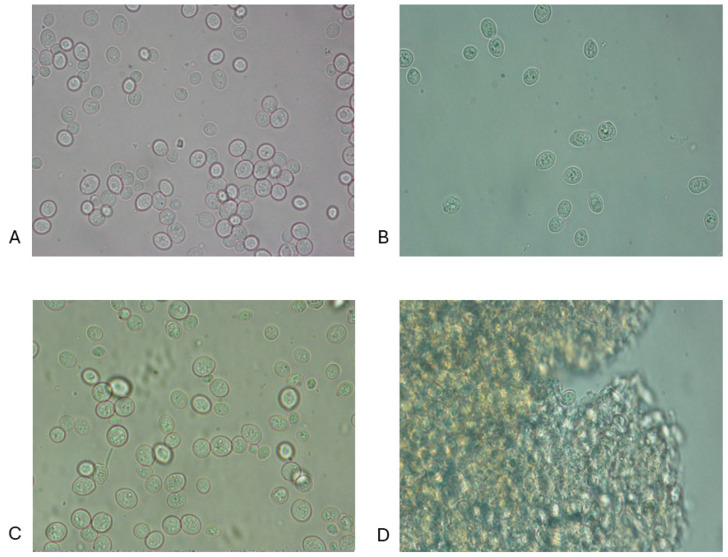
Microstructure (observation magn. 100×) of cell dispersions of *Saccharomyces cerevisiae* obtained after various treatments. (**A**) Not treated sample of BY; (**B**) not treated sample of BSY; (**C**) HPH+TT sample of BY; (**D**) HPH+TT sample of BSY.

**Figure 2:**
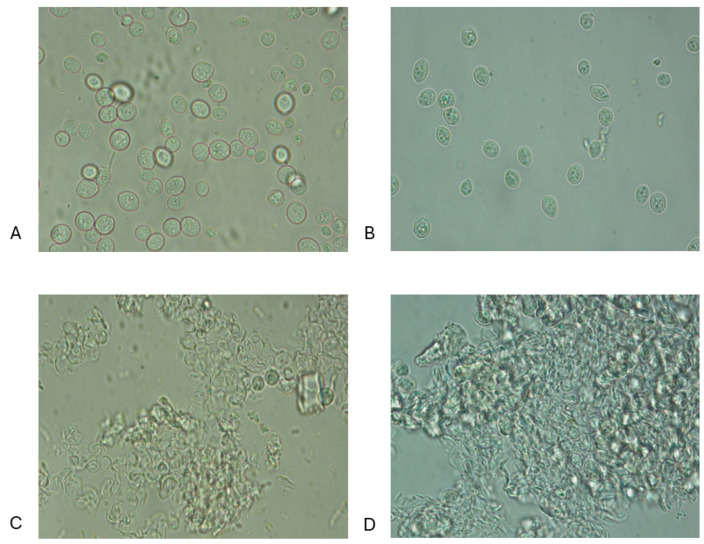
Microstructure (observation magn. 100×) of cell dispersions of *Saccharomyces cerevisiae* obtained after various treatments. (**A**) PEF sample of BY; (**B**) PEF sample of BSY; (**C**) HPH+PEF sample of BY; (**D**) HPH+PEF sample of BSY.

Microphotographs of the cell dispersions obtained by 100× and 40× objectives are reported below and as an example in Figure 1A–D and Figure 2A–D, which highlight the microphotographs (observation magn. 100×) of the dispersions of both baker’s yeast (BY) and brewer’s spent yeast (BSY), not treated (NT) and subjected to HPH+TT, PEF, and HPH+PEF treatments. The 40× magnification provided an overview suitable for assessing general cell integrity and detecting aggregation phenomena due to thermal and mechanical treatments [34]. The 100× magnification was chosen as a compromise between resolution and field of view, sufficient to observe morphological changes post-treatment. For other treatment groups, microphotographs are presented in the Supplementary File.

For the BY and BSY samples, similar behaviours were observed when subjected to the different treatments applied. In the case of the NT samples (Figure 1A), for which no treatments were applied, the cells were completely intact, with a well-defined, intact cell wall surrounding the cell cytoplasm. When the yeast dispersion was heated to 90 °C for only 20 min (TT) (Figures S2 and S11), the cells appeared slightly larger than the NT with slight turbidity due to a limited release of intracellular components. In this case, protein aggregation occurred inside the cell; therefore, the aggregates formed were narrow and thick and confined to the inner cellular space. Protein denaturation is ensured at the selected temperature (90 °C), as demonstrated in previous studies using whole cells of *S. cerevisiae*, and the peak denaturation temperature was about 66 °C for these cells [46]. 

When the same samples were subsequently treated with HPH, the turbidity increased, with a greater release of intracellular components. HPH (125 MPa) can destroy the yeast cell wall, favouring the release of the intracellular material into the surrounding medium [26,47], while heat treatment produces the inactivation of enzymatic reactions, protein denaturation, and unfolding of the triple helix of β-glucans present in the cell wall [15]. In the HPH samples (Figures S3 and S12), only a few undamaged cells were observed, and increased turbidity was visible, which can be attributed to the internal components released into the medium due to cell wall degradation. When heat treatment was applied before homogenisation (TT+HPH sample) (Figures S5 and S13), turbidity increased. In this case, protein denaturation occurred in the cytoplasm, and aggregation was limited to the cell wall. Subsequently, homogenisation promoted the breakdown of these aggregates, favouring their release into the medium. In addition, maintaining the suspension of yeast cells in autoclaves caused a partial loss of hot water-soluble cell wall structural polymers as a result of β-glucan extraction. Therefore, the order of treatment is very important, even if the treatments applied are identical (HPH+TT or TT+HPH). Applying HPH disturbs cells and releases proteins, which then aggregate differently when exposed to heat treatment (TT) (Figures S2 and S11). This arrangement is anticipated to produce more hydrated, open-structured protein aggregates because the proteins are exposed to an aqueous environment during heating, allowing them to interact more freely with water. 

In contrast, if thermal treatment is applied first, the proteins are denatured before mechanical forces are applied, which may result in less hydration and altered aggregate architectures. Thus, the sequence of treatments changes how the proteins cluster and interact with their environment, affecting the sample’s overall features [48]. Shear forces acting during homogenisation easily damaged the cells, whose walls were characterised by some tension and stiffness. It can be argued that after the heat treatment, the yeast cell wall lost its rigidity, and the subsequent mechanical breaking step helped to effectively remove the cell wall and cytosol components. However, ref. [49] explained that mild heat treatment mainly kills cells, which can lead to smaller, hardened cells, thus making subsequent mechanical breakdown less efficient. The selection of different treatment combinations, such as HPH+TT, TT+HPH, and PEF+HPH, allowed us to explore how the sequence of treatments influences cell lysis efficiency and intracellular component release. The results indicate that combinations like HPH+PEF produced a higher release of cell wall components compared with single treatments, suggesting that the disintegration effect is enhanced when mechanical and non-thermal treatments are applied in succession. A potential hypothesis to explain this effect is that PEF may initially create poration in the cell wall through electroporation, making the structure more susceptible to subsequent mechanical stress from HPH. This synergistic process could explain the greater degree of disintegration observed with combined treatments compared with that observed with PEF alone, which can increase poration but does not cause complete cell disintegration. 

In turn, [20,23,29,31] reported that the change in cell wall structure after autoclaving yeast cells, resulting in the loss of the mannoprotein layer, may enhance the mechanical disintegration process. Many studies have been published in recent years on the use of pulsed electric field (PEF) treatment as an alternate, non-thermal approach for extracting bioactive chemicals from microorganisms and plant tissues [37,40,50]. The electrical treatment causes electropermeabilisation or electroporation of the plasma membrane. Our results indicate that PEF treatment alone (Figure 2A,B and Figure S6) did not change the cellular structure. Cellular aggregations were observed when PEF treatment was combined with heat treatment (PEF+TT; TT+PEF) (Figures S7–S14; Figures S8–S15, respectively) or HPH treatment (HPH+PEF; PEF+HPH) (Figure 2C,D and Figure S9; Figures S10–S16, respectively). With the combination of PEF with HPH, a greater release of the cell wall components for both BY and BSY was observed. On the other hand, other authors reported that although PEF treatments can affect the cell wall poration, when applied alone they do not cause the complete lysis of yeast cells [31,51].

### 3.2. Dry Matter Content of Baker’s and Brewer’s Spent Saccharomyces cerevisiae Yeast 

The dry matter content, the evaluation was important to understanding the impact of the different treatments on yeast dispersions and their resulting supernatants post-centrifugation. As shown in Table 2, considering the dry matter data of the dispersions, in the case of BY, the dispersions were quite homogeneous amongst themselves, and regardless of the treatment applied, the dry matter ranged between 2.05 and 3.31%. Conversely, more variability was observed in the BSY samples, where significant differences were noted amongst the dispersions, with values ranging between 2.73 and 5.68%. This is certainly due to the imperfect homogeneity of the BSY biomass used in the trials. In fact, BSY can contain impurities such as spent grains or other residues from the brewing process, including hop residues, coagulated proteins, and other particles derived from the ingredients used in brewing. As expected, the dry matter content of the supernatants increased in all the treated samples compared with the NT ones. As shown in Table 2 dry matter in the supernatants varied depending on the yeast biomass considered and the treatment applied. Considering BY supernatants, the HPH-treated samples showed the highest dry matter content, ranging between 0.70 and 0.95%. In contrast, significantly lower values were evident in samples subjected to heat treatment only, PEF treatment only, and their combinations (TT: 0.30%; PEF: 0.11%; PEF+TT: 0.52%; TT+PEF: 0.54%). In the case of BSY biomasses, samples subjected to HPH showed similar dry matter content in the supernatants, ranging between 0.30 and 0.37%. Similarly, the PEF and PEF+TT samples showed a dry matter content of 0.24 and 0.23%, respectively, whereas PEF followed by HPH recorded a value of 0.39%. The highest dry matter content amongst the supernatants was in the HPH+PEF sample, at 0.89%, while other treated samples exhibited similar concentrations between 0.13% and 0.17%. 

These findings highlight the impact of different treatments on the release of cellular constituents into the extracellular environment, indicating different extraction efficiencies and alterations in material composition based on the treatment methodologies. As reported in the literature, heat can denature proteins and change cell structures; however, it may not be as effective at releasing cell constituents. PEF, while effective at permeabilizing cell membranes, is not generally sufficient alone to cause the rupture of the cell wall as thoroughly as HPH, as observed by [36] and confirmed by our results. HPH treatment, either alone or combined with other treatments, resulted in higher dry matter amounts in the supernatants, suggesting a potential for the increased release of intracellular components [36,37]. 

### 3.3. Evaluation of the Composition of Intracellular Material After Different Treatments

#### 3.3.1. Carbohydrate (CH) Concentration 

**Figure 3:**
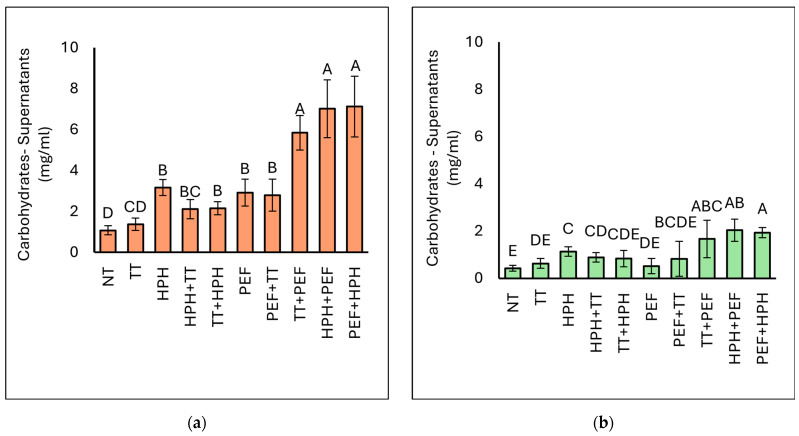
Concentration of carbohydrates (mg/mL) in the supernatants of BY (**a**) and BSY (**b**). NT represents the control sample. Different letters indicate significant differences (*p* < 0.05).

In Figure 3, the results regarding the carbohydrate concentration of the supernatants obtained after centrifugation of BY and BSY subjected to various treatments are reported. Considering the BY samples, the NT samples showed the lowest carbohydrate levels (1.08 mg/mL), as expected. All treatments led to a significant increase in carbohydrate content in the supernatants. The highest carbohydrate concentration was found in the PEF+HPH (7.13 mg/mL) and HPH+PEF (7.02 mg/mL) samples. However, the data indicate that the HPH and PEF treatments alone and in combination with TT increased the release of carbohydrates compared with the NT and TT samples. The results obtained indicate that the combination of PEF and HPH treatment favoured the release of intracellular carbohydrates from the yeast biomass. A similar trend was observed for BSY samples. The untreated sample showed the lowest carbohydrate value at 0.43 mg/mL, followed by the PEF sample (0.52 mg/mL) and the TT sample (0.63 mg/mL). Interestingly, the combination of HPH treatment with PEF treatment resulted in the highest release of carbohydrates into the cell supernatant (HPH+PEF: 2.04 mg/mL and PEF+HPH: 1.93 mg/mL). Nevertheless, the TT+PEF and HPH treatments also facilitated the release of more than 1.0 mg/mL of carbohydrates into the supernatant.

From the data obtained, it is clearly evident that the carbohydrate content in the supernatants is much higher in the BY samples compared with the BSY samples. This is due to the higher carbohydrate content in the native BY yeast (approximately 10 mg/mL) compared with the BSY yeast (approximately 2 mg/mL). Therefore, to better verify the effect of the treatments on the release of components, the dispersion index (DI) was determined, as shown in Table 3. The data reveal that the highest carbohydrate dispersible indices are achieved with the combined treatments of HPH+PEF and PEF+HPH in both BY and BSY yeasts, with generally higher values observed in BSY. Specifically, for BY yeast, the dispersible index for the HPH+PEF combination is 43.40%, while for PEF+HPH it is 44.07%. These values represent a significant increase compared with the individual treatments of HPH (19.59%) and PEF (18.02%). For BSY yeast, the dispersible index for the HPH+PEF combination reaches 70.37%, and for the PEF+HPH combination, it is 66.75%. This also marks a substantial increase compared with the individual treatments of HPH (39.25%) and PEF (17.93%). These results suggest a strong synergy between HPH and PEF, with a particularly pronounced effect in BSY, as demonstrated also by [52], who reported that combined PEF+HPH treatment can cause the release of carbohydrates from microalgal biomass even for 0.5% suspensions, resulting in higher carbohydrate contents with lower energy consumption for concentrated suspensions. 

#### 3.3.2. Protein Concentration 

**Figure 4:**
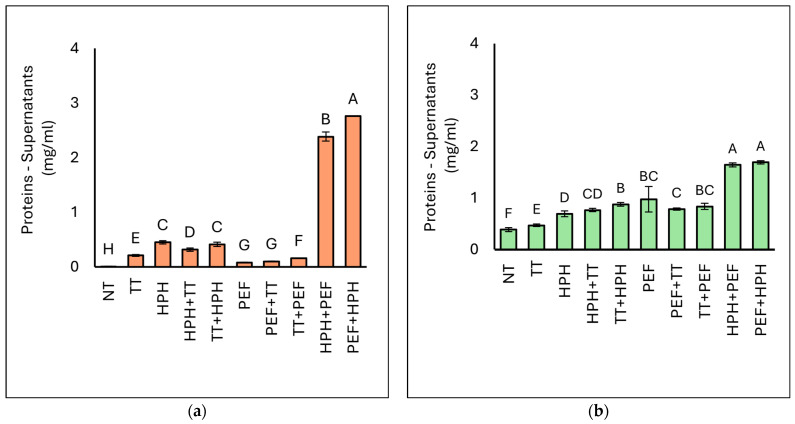
Concentration of proteins (mg/mL) of the supernatants of BY (**a**) and BSY (**b**). NT represents the control sample. Different letters indicate significant differences (*p* < 0.05).

The protein concentrations of the supernatants of BY and BSY are reported in Figure 4. The protein content in the native yeast suspension was approximately 3.0 mg/mL for baker’s yeast (BY) and 3.5 mg/mL for brewer’s spent yeast (BSY), measured per millilitres of yeast suspension. In contrast, the protein concentration in the supernatants ranged from 0.2 to 2.8 mg/mL for BY and from 0.39 to 1.69 mg/mL for BSY, depending on the specific treatment applied. As expected, a significant increase in protein content was observed in the supernatants of the treated samples compared with the untreated samples, in which the protein concentration was 0.01 and 0.39 mg/mL in BY and BSY, respectively. In both yeast biomasses considered, significantly higher protein levels were observed in the supernatants of the HPH+PEF and PEF+HPH samples. However, all the other treatments performed also resulted in a significant increase in protein in the supernatant compared with the control sample (NT) and the sample subjected to heat treatment alone (TT): this was observed mainly on BSY biomasses, while in the case of BY, the application of only PEF and its combination with heat treatment did not lead to a significant increase in protein concentration compared with the application of thermal treatment alone.

Our results indicate that heat treatment is not sufficient to obtain the release of protein from the intracellular components. From the obtained data, it appears that the combination of HPH, which promotes cell wall disruption, and PEF, which leads to electroporation phenomena, allows for the greater release of intracellular components. On the other hand, ref. [53] also reported that HPH significantly impacts the protein yield as it causes changes in yeast pH and viscosity through the micro-ionisation of cell debris. Similarly, ref. [28] integrated electrical treatment with HPH in a yeast suspension to enhance protein extraction; however, they observed that using PEF or high-voltage electrical discharges (HVEDs) alone did not achieve complete yeast cell rupture, even at settings of 40 kV/cm and 500 pulses. Interestingly, it has been highlighted that an increase in protein release below 10 kV/cm with few cell deaths could be observed, reinforcing our results [54]. The data relating to the dispersible index of proteins (Table 4) reveal that the highest protein dispersible indices are achieved with the combined treatments of HPH+PEF and PEF+HPH in both BY and BSY yeasts, with generally higher values observed in BSY. Specifically, for BY yeast, the dispersible index for the HPH+PEF combination is 78.85%, while for PEF+HPH it is 91.17%. For BSY yeast, the dispersible index for the HPH+PEF combination reaches 30.87%, and for the PEF+HPH combination, it is 31.78%. 

#### 3.3.3. Determination of β-(1,3)(1,6)-Glucans Content 

**Figure 5:**
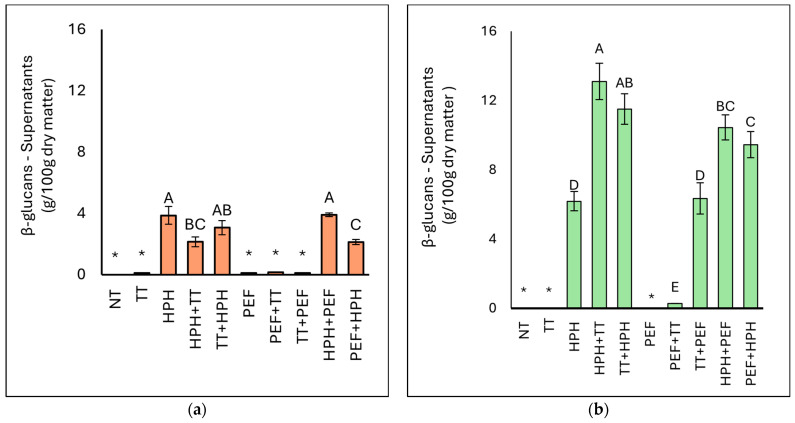
β-glucan content (g/100 g dry matter) of the supernatants of BY (**a**) and BSY (**b**). NT represents the control sample. Different letters indicate significant differences (*p* < 0.05). *: below the detection limit (1 g/100 g).

Figure 5 shows the β-glucan content of the supernatants of BY and BSY. The β-glucan content in the native yeasts was 9.50 g/100 g dry matter for BY and 9.89 g/100 g dry matter for BSY, respectively. The β-glucan content in the supernatants was dependent on the yeast biomass and the treatment applied [43]. All samples subjected to HPH (in combination with other treatments or not) were characterised by a significantly higher β-glucan content than the others. Considering BY biomasses, the HPH+PEF sample presented the highest β-glucan values of 3.90 g/100 g dry matter, followed by the HPH (3.86 g/100 g dry matter) and TT+HPH (3.07 g/100 g dry matter). On the contrary, the concentration of β-glucans in NT, TT, PEF, PEF+TT, and TT+PEF resulted below the detection limit (1 g/100 g). Considering BSY, the β-glucan concentration fell below the detectable limit in the NT, TT, and PEF samples. The HPH+TT and TT+HPH samples exhibited the highest β-glucan content in cellular supernatants, followed by the HPH+PEF and PEF+HPH samples, ranging between 13.10 g/100 g and 9.44 g/100 g of dry matter. The HPH and TT+PEF samples presented β-glucan concentrations above 6.0 g/100 g of dry matter, while the other samples showed significantly lower β-glucan concentrations compared with the latter. 

The biomass of *S. cerevisiae* yeast used by [55] to obtain cell walls via autolysis contained 17% of β-glucans. Homogenisation with glass beads was applied by [56] as well as by [57], but the cell wall preparations varied in the content of β-glucans (ca. 19–71%). The study of [25] helped to assess the application of HPH as a pretreatment prior to the process of autolysis of *S. cerevisiae* cells in order to enhance both the production of yeast extract and the valorisation of the solid residue of autolysis for β-glucan extraction. Analysing β-glucans and total protein in the crude insoluble autolysis residue revealed that HPH processing in conjunction with autolysis affected its composition. HPH treatment resulted in an increase in β-glucan content in the solid residue of autolysis. The lower contents of β-glucans might, in turn, result from the strain specificity of the applied biological material and the method of β-glucan analysis. It is also possible that the proteins and lipids present in the obtained cell wall preparations made difficult the hydrolytic action of the enzymes applied in the enzymatic test for β-glucan analysis. The mentioned compounds cause steric hindrance, and as a result, not all glycosidic bonds were hydrolysed; consequently, the β-glucan content would be underestimated. The data related to the dispersible index of β-glucans (Table 5) indicate that the highest β-glucans dispersible index is achieved with the combined treatments of HPH+PEF in BSY yeast, while for BY yeast, the individual treatment of HPH shows significant dispersion. Generally, BSY shows higher β-glucans dispersible indices than BY. Specifically, for BY yeast, the highest dispersible index is achieved with HPH (14.92%), followed by HPH+TT (8.26%) and TT+HPH (11.88%). The combination HPH+PEF results in a dispersible index of 11.19%, whereas PEF+HPH shows a dispersible index of 6.74%. For BSY yeast, the highest dispersible index is observed with HPH+PEF (23.19%), followed by HPH+TT (11.70%) and PEF+HPH (9.23%). The individual treatment of HPH results in a dispersible index of 5.66%. The analysis of the data reveals that both BY and BSY yeasts show varying degrees of β-glucans dispersion when subjected to different physical treatments (Table 5). The most effective treatment for BSY yeast is the combination of HPH+PEF, which results in the highest dispersion index (23.19%). In contrast, BY yeast achieves its highest dispersion index with the individual HPH treatment (14.92%). 

The significant differences (*p* < 0.05) in dispersible indices between BY and BSY can be attributed to several factors. Firstly, BSY typically contains a higher concentration of residual β-glucans due to the cell wall structure being less intact after fermentation, making it more amenable to extraction processes. Secondly, the composition and arrangement of polysaccharides in BSY may differ from that of BY, resulting in varied interactions during treatment. Additionally, the presence of different proteins and other cellular components in BSY can influence the efficacy of the physical treatments, allowing for a more effective release of β-glucans compared with BY. When examining the effect of single treatments, thermal treatment (TT) results in minimal β-glucan dispersion for both yeasts, with BY showing a slight increase to 0.26% and BSY remaining at 0.00%. The combination treatments of HPH+TT and TT+HPH show increased dispersion indices compared with individual treatments. For BY, HPH+TT achieves 8.26% and TT+HPH reaches 11.88%. For BSY, HPH+TT results in 11.70% and TT+HPH in 8.54%. The combination of HPH+PEF shows significant efficacy, with indices of 11.19% in BY and 23.19% in BSY, indicating a strong synergistic effect. The combination of HPH+PEF is particularly effective, especially for BSY yeast, suggesting that these techniques can be utilised to substantially enhance β-glucan dispersion in industrial applications. The results clearly indicate that the order of treatments and the type of yeast play crucial roles in determining the overall effectiveness of the dispersion process. As ref. [58] noted, yeast dispersions are multi-component systems in which more than one hydrocolloid (usually protein and polysaccharide) is present. These systems are capable of forming the continuous and cohesive network needed to produce films. Therefore, it is critical to release the right amounts of proteins and carbohydrates into the medium so that they can be used to form polymeric compounds. The visible cell wall fragments of the damaged cells show that cell degradation occurred simultaneously. Similarly, in the studies of [59,60], the formation of aggregates of yeast cell wall polymers was observed, suggesting the presence of particulate hydrophobic β-glucans. In the cited experiments, however, the aggregates were formed after alkali extraction of the β-glucans from the cell wall.

#### 3.3.4. Determination of Mannoprotein/Mannose Content 

**Figure 6:**
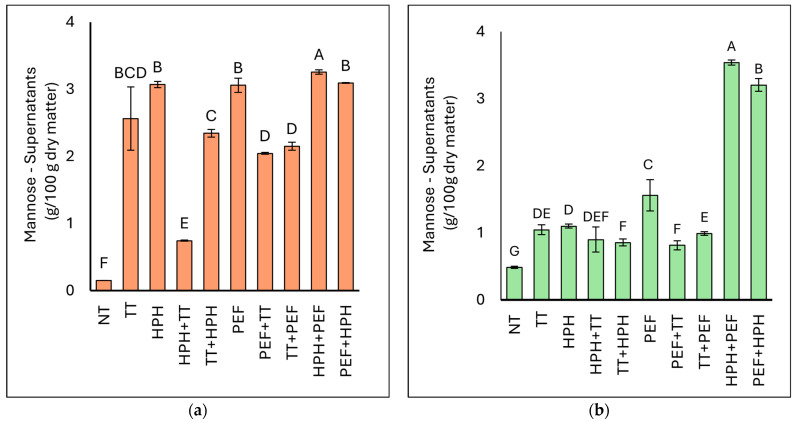
Mannose content (g/100 g dry matter) of the supernatants of BY (**a**) and BSY (**b**). NT represents the control sample. Different letters indicate significant differences (*p* < 0.05).

The mannose content in the supernatants of BY and BSY is reported in Figure 6. The mannose content in the supernatants of the NT samples was found to be 0.15 g/100 g dry matter in BY samples, and it was 0.49 g/100 g in the BSY samples. The effect of the treatments on the mannose content of the samples was dependent on the type of yeast. In the case of BY, all treatments, including just thermal treatment, led to a high presence of mannose in the supernatants. In contrast, for BSY, although all treatments resulted in increased mannose levels in the supernatants, the combination of HPH and PEF led to the highest release of mannose.

The data illustrated in Table 6 indicate that the highest mannoprotein dispersible indices are achieved with the combined treatments of HPH+PEF and PEF+HPH, particularly in BSY yeast. Overall, BY yeast shows higher mannoprotein dispersion indices compared with BSY yeast. For BY yeast, the highest dispersible indices are observed with HPH (58.02%) and the combined treatments HPH+PEF (45.75%) and PEF+HPH (48.03%). This suggests that high-pressure homogenisation is highly effective at disrupting the yeast cell wall and releasing mannoproteins. Thermal treatment (TT) alone shows a significant increase in the dispersion index for BY yeast (30.58%) but a much lower effect on BSY yeast (1.05%). This indicates that BY yeast is more susceptible to thermal treatment in terms of mannoprotein release. For BSY yeast, the combination of HPH+PEF results in the highest dispersion index (38.23%), followed by PEF+HPH (27.52%). 

This highlights the effectiveness of these combined treatments in enhancing mannoprotein release, although the overall dispersion indices in BSY are lower compared with BY. It was observed by [45] that PEF-treated yeast cells could provide 10 times more mannose concentration compared with the control. Electroporation-induced plasmolysis of the vacuoles containing hydrolytic enzymes can cause the liberation of mannoproteins in the supernatants, as described by [35]. A maximum of 60% of total mannose has been extracted and reported in the literature from yeast upon sequential extraction [61]. In accordance with the literature, we observed a maximum extraction of ~60% of mannose from the supernatants of BY with HPH treatment and a maximum extraction of ~40% of mannose from the supernatants of BSY with HPH+PEF treatment. 

## 4. Conclusions

This study demonstrates that combining high-pressure homogenisation (HPH) and pulsed electric fields (PEFs) significantly enhances β-glucan extraction from bakery yeast (BY) and brewer’s spent yeast (BSY), offering a non-thermal approach beyond conventional methods. Unlike traditional extraction techniques, the integration of HPH and PEF optimises cell wall disruption, improves yields, and supports scalability, making it a more sustainable and environmentally friendly solution. The combined HPH+PEF treatment yielded the highest releases of carbohydrates (7.02 mg/mL in BY and 2.04 mg/mL in BSY), proteins (2.39 mg/mL in BY and 1.64 mg/mL in BSY), β-glucans (3.90 g/100 g dry matter in BY and 10.44 g/100 g dry matter in BSY), and mannoproteins (3.26 g/100 g dry matter in BY and 3.54 g/100 g dry matter in BSY). Industrial relevance is evident in utilizing BSY, a brewing by-product, as a valuable raw material for biodegradable food packaging, aligning with circular economy goals and reducing reliance on petrochemical plastics. Additionally, the bioactive properties of the extracted β-glucans make them suitable for applications in cosmetics and pharmaceuticals, adding commercial value beyond packaging. Although the PEF treatment conditions (1.5 kV/cm, 0.012 kJ/kg) in this study are optimised for cell disintegration, they are lower than the threshold typically required to disrupt yeast cell membranes (≥ 10 kV/cm, ≥ 50 kJ/kg). However, the combination of PEF with HPH significantly enhanced β-glucan release, suggesting a synergistic effect. Future studies should consider exploring higher PEF intensities to further improve yeast cell wall disruption. This work advances sustainable biopolymer production and provides a pathway for waste valorisation, with future research focusing on industrial-scale implementation and exploring additional applications for these biopolymers in creating value-added products across multiple sectors. 

## Figures and Tables

**Table 1:** Types of different treatments performed to obtain samples from BY and BSY biomasses.

Treatments Description	Performed Treatments
NT	Not treated
TT	Heat treatment (TT: 90 °C for 20 min)
HPH	3 cycles of HPH at a pressure of 125 MPa
HPH+TT	3 cycles of HPH at a pressure of 125 MPa followed by heat treatment (TT: 90 °C for 20 min)
TT+HPH	Heat treatment (TT: 90 °C for 20 min) followed by 3 cycles of HPH at a pressure of 125 MPa
PEF	Pulsed electric field treatment (1.5 kV/cm—45 pulses)
PEF+TT	Pulsed electric field treatment (1.5 kV/cm—45 pulses) followed by heat treatment (TT: 90 °C for 20 min)
TT+PEF	Heat treatment (TT: 90 °C for 20 min) followed by a pulsed electric field treatment (1.5 kV/cm—45 pulses)
HPH+PEF	3 cycles of HPH at a pressure of 125 MPa followed by a pulsed electric field treatment (1.5 kV/cm—45 pulses)
PEF+HPH	3 cycles of HPH at a pressure of 125 MPa followed by a pulsed electric field treatment (1.5 kV/cm—45 pulses)

**Table 2:** Dry matter content (%) of dispersions and supernatants of BY and BSY obtained after the application of the different treatments. NT represents the control sample. Different letters for each yeast biomass in the same column indicate significant differences amongst mean observations (*p* < 0.05).

Yeast Biomass	Treatment	Dispersions ± DS (%)	Supernatants ± DS (%)
BY	NT	2.82 ± 0.25 ^ab^	0.04 ± 0.03 ^c^
	TT	2.70 ± 0.49 ^ab^	0.30 ± 0.12 ^ab^
	HPH	2.68 ± 0.21 ^ab^	0.95 ± 0.02 ^a^
	HPH+TT	3.31 ± 0.71 ^a^	0.94 ± 0.02 ^a^
	TT+HPH	2.81 ± 0.63 ^ab^	0.95 ± 0.02 ^a^
	PEF	2.05 ± 0.56 ^bc^	0.11 ± 0.01 ^c^
	PEF+TT	2.59 ± 0.01 ^b^	0.52 ± 0.09 ^ab^
	TT+PEF	2.59 ± 0.01 ^b^	0.54 ± 0.04 ^ab^
	HPH+PEF	2.12 ± 0.08 ^bc^	0.70 ± 0.23 ^ab^
	PEF+HPH	2.24 ± 0.01 ^bc^	0.78 ± 0.20 ^ab^
BYS	NT	3.08 ± 0.11 ^d^	0.01 ± 0.00 ^g^
	TT	4.38 ± 0.83 ^abc^	0.05 ± 0.01 ^f^
	HPH	4.09 ± 1.26 ^abcd^	0.37 ± 0.07 ^bc^
	HPH+TT	2.73 ± 0.28 ^d^	0.36 ± 0.03 ^b^
	TT+HPH	3.31 ± 0.36 ^cd^	0.30 ± 0.02 ^c^
	PEF	3.12 ± 0.07 ^d^	0.24 ± 0.03 ^d^
	PEF+TT	3.43 ± 0.50 ^cd^	0.23 ± 0.01 ^d^
	TT+PEF	5.50 ± 0.39 ^a^	0.13 ± 0.01 ^e^
	HPH+PEF	3.96 ± 0.78 ^bcd^	0.89 ± 0.00 ^a^
	PEF+HPH	5.68 ± 0.57 ^a^	0.39 ± 0.10 ^bc^

**Table 3:** Dispersible index of carbohydrates for BY and BSY. Different superscript letters indicate significant differences (*p* < 0.05) along each column.

Samples	Dispersible Index of BY (%)	Dispersible Index of BSY (%)
NT	6.67 ± 1.91 ^e^	14.76 ± 5.99 ^d^
TT	8.54 ± 2.61 ^de^	21.82 ± 10.32 ^cd^
HPH	19.59 ± 3.38 ^c^	39.25 ± 9.78 ^bc^
HPH+TT	13.09 ± 4.08 ^cd^	30.75 ± 9.74 ^cd^
TT+HPH	13.39 ± 2.80 ^cd^	28.88 ± 16.95 ^bcd^
PEF	18.02 ± 5.75 ^cd^	17.93 ± 15.79 ^cd^
PEF+TT	17.35 ± 6.83 ^cd^	28.44 ± 26.41 ^bcd^
TT+PEF	36.16 ± 7.34 ^a^	57.55 ± 34.23 ^abc^
HPH+PEF	43.40 ± 12.40 ^a^	70.37 ± 22.71 ^ab^
PEF+HPH	44.07 ± 12.98 ^a^	66.75 ± 10.37 ^a^

**Table 4:** Dispersible index of proteins for BY and BSY. Different superscript letters indicate significant differences (*p* < 0.05) along each column.

Samples	Dispersible Index of BY (%)	Dispersible Index of BSY (%)
NT	0.31 ± 0.17 ^h^	7.30 ± 1.06 ^d^
TT	7.00 ± 0.68 ^e^	8.81 ± 0.66 ^d^
HPH	14.94 ± 1.40 ^c^	13.05 ± 1.49 ^c^
HPH+TT	10.55 ± 1.38 ^d^	14.37 ± 0.82 ^bc^
TT+HPH	13.70 ± 1.77 ^cd^	16.49 ± 0.93 ^b^
PEF	2.73 ± 0.11 ^g^	18.37 ± 6.54 ^bc^
PEF+TT	3.42 ± 0.09 ^g^	14.75 ± 0.62 ^bc^
TT+PEF	5.30 ± 0.09 ^f^	15.70 ± 1.58 ^bc^
HPH+PEF	78.85 ± 3.99 ^b^	30.87 ± 1.03 ^a^
PEF+HPH	91.17 ± 0.14 ^a^	31.78 ± 0.82 ^a^

**Table 5:** Dispersible index of β-glucans for BY and BSY. Different superscript letters indicate significant differences (*p* < 0.05) along each column.

Samples	Dispersible Index of BY (%)	Dispersible Index of BSY (%)
NT	0.00	0.00
TT	0.26 ± 0.01 ^f^	0.00
HPH	14.92 ± 3.17 ^a^	5.66 ± 0.72 ^d^
HPH+TT	8.26 ± 1.75 ^cd^	11.70 ± 1.33 ^b^
TT+HPH	11.88 ± 2.52 ^abc^	8.54 ± 0.93 ^c^
PEF	0.05 ± 0.01 ^g^	0.00
PEF+TT	0.32 ± 0.01 ^e^	0.16 ± 0.01 ^f^
TT+PEF	0.22 ± 0.01 ^f^	2.06 ± 0.41 ^e^
HPH+PEF	11.19 ± 0.46 ^b^	23.19 ± 2.29 ^a^
PEF+HPH	6.74 ± 0.79 ^d^	9.23 ± 1.04 ^c^

**Table 6:** Dispersible index of mannoproteins in BY and BSY. Different superscript letters indicate significant differences (*p* < 0.05) along each column.

Samples	Dispersible Index of BY (%)	Dispersible Index of BSY (%)
NT	0.13 ± 0.01 ^i^	0.32 ± 0.01 ^h^
TT	30.58 ± 3.99 ^d^	1.05 ± 0.10 ^g^
HPH	58.02 ± 1.24 ^a^	8.87 ± 0.34 ^c^
HPH+TT	14.07 ± 0.28 ^g^	7.06 ± 2.07 ^cd^
TT+HPH	44.46 ± 1.53 ^c^	5.58 ± 0.48 ^d^
PEF	6.56 ± 0.31 ^h^	8.29 ± 1.75 ^c^
PEF+TT	21.14 ± 0.23 ^f^	4.09 ± 0.48 ^e^
TT+PEF	23.07 ± 0.94 ^e^	2.83 ± 0.10 ^f^
HPH+PEF	45.75 ± 0.62 ^c^	38.23 ± 1.00 ^a^
PEF+HPH	48.03 ± 0.11 ^b^	27.52 ± 1.17 ^b^

## Data Availability

The original contributions presented in the study are included in the article; further inquiries can be directed to the corresponding author.

## Supplementary Materials

The following supporting information can be downloaded at: https://www.mdpi.com/article/10.3390/microorganisms12122596/s1, Figure S1: Observation of NT samples (magn.40×); Figure S2: Observation of TT samples (magn.40×); Figure S3: Observation of HPH samples (magn.40×); Figure S4: Observation of HPH+TT samples (magn.40×); Figure S5: Observation of TT+HPH samples (magn.40×); Figure S6: Observation of PEF samples (magn.40×); Figure S7: Observation of PEF+TT samples (magn. 40×); Figure S8: Observation of TT+PEF samples (magn.40×); Figure S9: Observation of HPH+PEF samples (magn.40×); Figure S10: Observation of PEF+HPH samples (magn.40×); Figure S11: Observation of TT samples (magn.100×); Figure S12: Observation of HPH samples (magn.100×); Figure S13: Observation of TT+HPH samples (magn.100×); Figure S14: Observation of PEF+TT samples (magn.100×); Figure S15: Observation of TT+PEF samples (magn.100×); Figure S16: Observation of PEF+HPH samples (magn.100×).

## References

[1] Crippa M., Solazzo E., Guizzardi D., Monforti-Ferrario F., Tubiello F.N., Leip A. (2021). Food Systems Are Responsible for a Third of Global Anthropogenic GHG Emissions. Nat. Food.

[2] Jamwal V., Mittal A., Dhaundiyal A. (2023). Valorization of Agro-Industrial Waste in Composite Films for Sustainable Packaging Applications. Mater. Today Proc..

[3] Tardy B.L., Richardson J.J., Greca L.G., Guo J., Bras J., Rojas O.J. (2023). Advancing Bio-Based Materials for Sustainable Solutions to Food Packaging. Nat. Sustain..

[4] Peltzer M., Delgado J., Salvay A., Wagner J. (2018). β-Glucan, a Promising Polysaccharide for Bio-Based Films Developments for Food Contact Materials and Medical Applications. Curr. Org. Chem..

[5] Ferreira I.M.P.L.V.O., Pinho O., Vieira E., Tavarela J.G. (2010). Brewer’s Saccharomyces Yeast Biomass: Characteristics and Potential Applications. Trends Food Sci. Technol..

[6] França R.A.D., Rosa A.C.F.D.S., Braz C.J.D.F., Barbosa R., Alves T.S. (2024). Development of Mulch Films from Biodegradable Polymer and Agro-Industrial Waste. Polímeros.

[7] Hejna A. (2022). More than Just a Beer—The Potential Applications of by-Products from Beer Manufacturing in Polymer Technology. Emergent. Mater..

[8] Rachwał K., Waśko A., Gustaw K., Polak-Berecka M. (2020). Utilization of Brewery Wastes in Food Industry. PeerJ.

[9] Cottet C., Ramirez-Tapias Y.A., Delgado J.F., de la Osa O., Salvay A.G., Peltzer M.A. (2020). Biobased Materials from Microbial Biomass and Its Derivatives. Materials.

[10] Delgado J.F., Salvay A.G., de la Osa O., Wagner J.R., Peltzer M.A. (2021). Impact of the Film-Forming Dispersion PH on the Properties of Yeast Biomass Films. J. Sci. Food Agric..

[11] Faustino M., Durão J., Pereira C.F., Oliveira A.S., Pereira J.O., Pereira A.M., Ferreira C., Pintado M.E., Carvalho A.P. (2022). Comparative Analysis of Mannans Extraction Processes from Spent Yeast *Saccharomyces cerevisiae*. Foods.

[12] Avramia I., Amariei S. (2021). Spent Brewer’s Yeast as a Source of Insoluble β-Glucans. Int. J. Mol. Sci..

[13] Tam T.M., Duy N.Q., Minh N.P., Dao D.T.A. (2013). Optimization of Βeta-Glucan Extraction from Waste Brewer’s Yeast *Saccharomyces cerevisiae* Using Autolysis, Enzyme, Ultrasonic and Combined Enzyme—Ultrasonic Treatment. Am. J. Res. Commun..

[14] Du B., Meenu M., Liu H., Xu B. (2019). A Concise Review on the Molecular Structure and Function Relationship of β-Glucan. Int. J. Mol. Sci..

[15] Novák M., Synytsya A., Gedeon O., Slepička P., Procházka V., Synytsya A., Blahovec J., Hejlová A., Čopíková J. (2012). Yeast β(1-3),(1-6)-d-Glucan Films: Preparation and Characterization of Some Structural and Physical Properties. Carbohydr. Polym..

[16] Razzaq H.A.A., Pezzuto M., Santagata G., Silvestre C., Cimmino S., Larsen N., Duraccio D. (2016). Barley β-Glucan-Protein Based Bioplastic Film with Enhanced Physicochemical Properties for Packaging. Food Hydrocoll..

[17] Sherafatkhah Azari S., Alizadeh A., Roufegarinejad L., Asefi N., Hamishehkar H. (2021). Preparation and Characterization of Gelatin/β-Glucan Nanocomposite Film Incorporated with ZnO Nanoparticles as an Active Food Packaging System. J. Polym. Environ..

[18] Podpora B., Świderski F., Sadowska A., Piotrowska A., Rakowska R. (2015). Spent Brewer’s Yeast Autolysates as a New and Valuable Component of Functional Food and Dietary Supplements. J. Food Process. Technol..

[19] Chakka A.K., Babu A.S. (2022). Bioactive Compounds of Winery By-Products: Extraction Techniques and Their Potential Health Benefits. Appl. Food Res..

[20] Liu X., Wang Q., Cui S., Liu H. (2008). A New Isolation Method of β-d-Glucans from Spent Yeast *Saccharomyces cerevisiae*. Food Hydrocoll..

[21] Utama G.L., Oktaviani L., Balia R.L., Rialita T. (2023). Potential Application of Yeast Cell Wall Biopolymers as Probiotic Encapsulants. Polymers.

[22] Patrignani F., Lanciotti R. (2016). Applications of High and Ultra High Pressure Homogenization for Food Safety. Front. Microbiol..

[23] Liu D., Ding L., Sun J., Boussetta N., Vorobiev E. (2016). Yeast Cell Disruption Strategies for Recovery of Intracellular Bio-Active Compounds—A Review. Innov. Food Sci. Emerg. Technol..

[24] Gottardi D., Siroli L., Braschi G., Rossi S., Ferioli F., Vannini L., Patrignani F., Lanciotti R. (2021). High-Pressure Homogenization and Biocontrol Agent as Innovative Approaches Increase Shelf Life and Functionality of Carrot Juice. Foods.

[25] Dimopoulos G., Tsantes M., Taoukis P. (2020). Effect of High Pressure Homogenization on the Production of Yeast Extract via Autolysis and Beta-Glucan Recovery. Innov. Food Sci. Emerg. Technol..

[26] Spiden E.M., Scales P.J., Kentish S.E., Martin G.J.O. (2013). Critical Analysis of Quantitative Indicators of Cell Disruption Applied to *Saccharomyces cerevisiae* Processed with an Industrial High Pressure Homogenizer. Biochem. Eng. J..

[27] Dimopoulos G., Stefanou N., Andreou V., Taoukis P. (2018). Effect of Pulsed Electric Fields on the Production of Yeast Extract by Autolysis. Innov. Food Sci. Emerg. Technol..

[28] Liu D., Lebovka N.I., Vorobiev E. (2013). Impact of Electric Pulse Treatment on Selective Extraction of Intracellular Compounds from *Saccharomyces cerevisiae* Yeasts. Food Bioproc. Tech..

[29] Shynkaryk M.V., Lebovka N.I., Lanoisellé J.-L., Nonus M., Bedel-Clotour C., Vorobiev E. (2009). Electrically-Assisted Extraction of Bio-Products Using High Pressure Disruption of Yeast Cells (*Saccharomyces cerevisiae*). J. Food Eng..

[30] Martínez J.M., Delso C., Álvarez I., Raso J. (2020). Pulsed Electric Field-Assisted Extraction of Valuable Compounds from Microorganisms. Compr. Rev. Food Sci. Food Saf..

[31] Ganeva V., Galutzov B., Teissie J. (2014). Evidence That Pulsed Electric Field Treatment Enhances the Cell Wall Porosity of Yeast Cells. Appl. Biochem. Biotechnol..

[32] Bzducha-Wróbel A., Błażejak S., Kawarska A., Stasiak-Różańska L., Gientka I., Majewska E. (2014). Evaluation of the Efficiency of Different Disruption Methods on Yeast Cell Wall Preparation for β-Glucan Isolation. Molecules.

[33] Paniagua-Martínez I., Ramírez-Martínez A., Serment-Moreno V., Rodrigues S., Ozuna C. (2018). Non-Thermal Technologies as Alternative Methods for *Saccharomyces cerevisiae* Inactivation in Liquid Media: A Review. Food Bioproc. Tech..

[34] Marín-Sánchez J., Berzosa A., Álvarez I., Sánchez-Gimeno C., Raso J. (2024). Selective Extraction of Biomolecules from *Saccharomyces cerevisiae* Assisted by High-Pressure Homogenization, Pulsed Electric Fields, and Heat Treatment: Exploring the Effect of Endogenous Enzymes. LWT.

[35] Berzosa A., Delso C., Sanz J., Sánchez-Gimeno C., Raso J. (2023). Sequential Extraction of Compounds of Interest from Yeast Biomass Assisted by Pulsed Electric Fields. Front. Bioeng. Biotechnol..

[36] Carullo D., Abera B.D., Scognamiglio M., Donsì F., Ferrari G., Pataro G. (2022). Application of Pulsed Electric Fields and High-Pressure Homogenization in Biorefinery Cascade of *C. vulgaris* Microalgae. Foods.

[37] Carullo D., Abera B.D., Casazza A.A., Donsì F., Perego P., Ferrari G., Pataro G. (2018). Effect of Pulsed Electric Fields and High Pressure Homogenization on the Aqueous Extraction of Intracellular Compounds from the Microalgae *Chlorella vulgaris*. Algal Res..

[38] Delgado J.F., Sceni P., Peltzer M.A., Salvay A.G., de la Osa O., Wagner J.R. (2016). Development of Innovative Biodegradable Films Based on Biomass of *Saccharomyces cerevisiae*. Innov. Food Sci. Emerg. Technol..

[39] DuBois M., Gilles K.A., Hamilton J.K., Rebers P.T., Smith F. (1956). Colorimetric Method for Determination of Sugars and Related Substances. Anal. Chem..

[40] Ganeva V., Angelova B., Galutzov B., Goltsev V., Zhiponova M. (2020). Extraction of Proteins and Other Intracellular Bioactive Compounds From Baker’s Yeasts by Pulsed Electric Field Treatment. Front. Bioeng. Biotechnol..

[41] Gottardi D., Ciccone M., Siroli L., Lanciotti R., Patrignani F. (2022). Use of *Yarrowia lipolytica* to obtain fish waste functional hydrolysates rich in flavoring compounds. Fermentation.

[42] Vaithanomsat P., Boonlum N., Trakunjae C., Apiwatanapiwat W., Janchai P., Boondaeng A., Phalinphattharakit K., Nimitkeatkai H., Jarerat A. (2022). Functionality of Yeast β-Glucan Recovered from *Kluyveromyces marxianus* by Alkaline and Enzymatic Processes. Polymers.

[43] Varelas V., Liouni M., Calokerinos A.C., Nerantzis E.T. (2016). An Evaluation Study of Different Methods for the Production of β-D-Glucan from Yeast Biomass. Drug Test Anal..

[44] Quirós M., Gonzalez R., Morales P. (2012). A Simple Method for Total Quantification of Mannoprotein Content in Real Wine Samples. Food. Chem..

[45] Martínez J.M., Delso C., Maza M.A., Álvarez I., Raso J. (2019). Pulsed Electric Fields Accelerate Release of Mannoproteins from *Saccharomyces cerevisiae* during Aging on the Lees of Chardonnay Wine. Food Res. Int..

[46] Otero M.A., Wagner J.R., Vasallo M.C., García L., Añón M.C. (2000). Thermal Behavior and Hydration Properties of Yeast Proteins from *Saccharomyces cerevisiae* and *Kluyveromyces fragilis*. Food Chem..

[47] Ekpeni L.E.N. (2015). Investigation and Disruption of Baker’s Yeast/*Chlorella vulgaris* in High-Pressure Homogenizer (HPH) to Improve Cost-Effective Protein Yield. Ph.D. Thesis.

[48] Huang Z., Zhang J., Zhang G., Gao F., Bi C. (2023). The Impact of High-Pressure Homogenization and Thermal Processing on the Functional Properties of De-Fatted Chickpea Flour Dispersion. Foods.

[49] Middelberg A.P.J. (1995). Process-Scale Disruption of Microorganisms. Biotechnol. Adv..

[50] Martínez J.M., Cebrián G., Álvarez I., Raso J. (2016). Release of Mannoproteins during *Saccharomyces cerevisiae* Autolysis Induced by Pulsed Electric Field. Front. Microbiol..

[51] Ganeva V., Galutzov B., Teissié J. (2003). High Yield Electroextraction of Proteins from Yeast by a Flow Process. Anal Biochem..

[52] Zhang R. (2020). Impact of Emerging Technologies on the Cell Disruption and Fractionation of Microalgal Biomass. Ph.D. Thesis.

[53] Balasundaram B., Harrison S.T.L. (2008). Influence of the Extent of Disruption of Bakers’ Yeast on Protein Adsorption in Expanded Beds. J. Biotechnol..

[54] Gentès M.-C., Caron A., Champagne C.P. (2022). Potential Applications of Pulsed Electric Field in Cheesemaking. Int J. Dairy Technol..

[55] Suphantharika M., Khunrae P., Thanardkit P., Verduyn C. (2003). Preparation of Spent Brewer’s Yeast B-Glucans with a Potential Application as an Immunostimulant for Black Tiger Shrimp, Penaeus Monodon. Bioresour. Technol..

[56] Dallies N., François J., Paquet V. (1998). A New Method for Quantitative Determination of Polysaccharides in the Yeast Cell Wall. Application to the Cell Wall Defective Mutants of *Saccharomyces cerevisiae*. Yeast.

[57] Marinescu G., Stoicescu A. (2009). Researches concerning the preparation of spent brewer’s yeast β-glucans. J. Agroaliment. Process. Technol..

[58] Giancone T., Torrieri E., Masi P., Michon C. (2009). Protein–Polysaccharide Interactions: Phase Behaviour of Pectin–Soy Flour Mixture. Food Hydrocoll..

[59] Hunter Jr K.W., Gault R.A., Berner M.D. (2002). Preparation of Microparticulate Β-glucan from *Saccharomyces cerevisiae* for Use in Immune Potentiation. Lett. Appl. Microbiol..

[60] Zechner-Krpan V., Petravić-Tominac V., Galović P., Galović V., Filipović-Grčić J., Srečec S. (2010). Application of Different Drying Methods on β-Glucan Isolated from Spent Brewer’s Yeast Using Alkaline Procedure. Agric. Conspec. Sci..

[61] Ganeva V., Kranz A. (2023). Selective Extraction of Recombinant Membrane Proteins from Hansenula Polymorpha by Pulsed Electric Field and Lytic Enzyme Pretreatment. Microb. Cell. Fact..

