# Supplementary material for: Release of Biopolymers from Saccharomyces cerevisiae Biomass Through Thermal and Non-Thermal Technologies
Source: Microorganisms. 2024 Dec 15;12(12):2596. doi: 10.3390/microorganisms12122596 (PMC11677850; doi:10.3390/microorganisms12122596)
Supplement: Supplementary file 1 [file microorganisms-12-02596-s001.zip › microorganisms-3343782-supplementary.pdf]

Supplementary Information

Release of Biopolymers from *Saccharomyces cerevisiae* Biomass Through Thermal and Non-Thermal Technologies

Marianna Ciccone[1]*, Muhammad Rehan Khan[1], Junior Bernardo Molina Hernandez[1], Joel Armando Njieukam[1], Lorenzo Siroli[1,2], Davide Gottardi[1,2], Rosalba Lanciotti[1,2], Pietro Rocculi[1,2] and Francesca Patrignani[1,2]

[1] Department of Agricultural and Food Sciences, Campus of Food Science, Alma Mater Studiorum, University of Bologna, Piazza Goidanich 60, 47521 Cesena, Italy

[2] Interdepartmental Centre for Agri-Food Industrial Research, Campus of Food Science, Alma Mater Studiorum, University of Bologna, Via Quinto Bucci 336, 47521 Cesena, Italy

* Correspondence: marianna.ciccone2@unibo.it

Saccharomyces cerevisiae: Observation (magnification 40×)

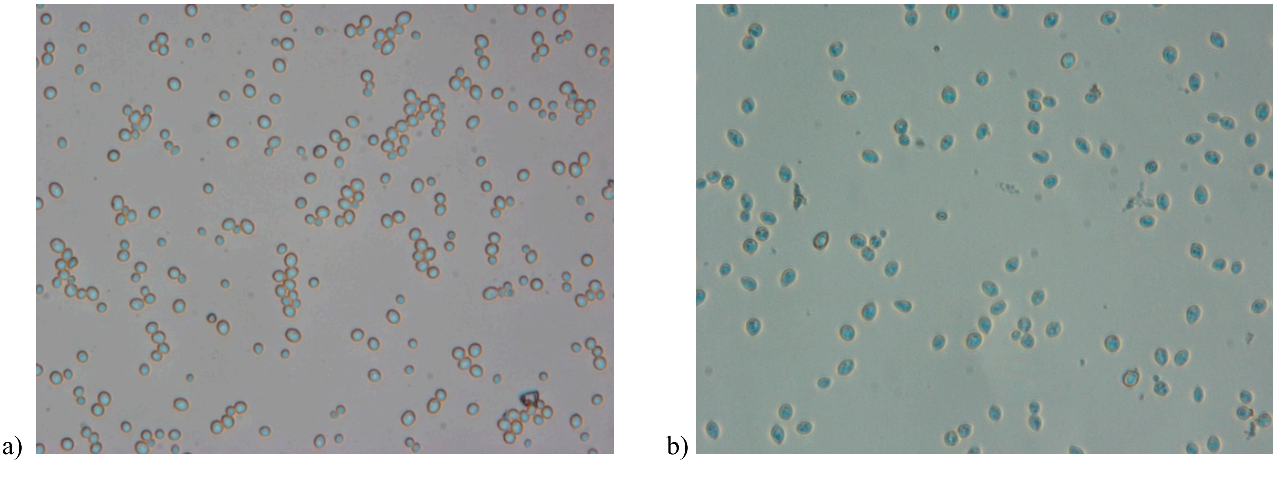

Figure S1: Observation of NT samples (magn.40×)
a) Baker’s commercial yeast; b) Brewer’s spent yeast

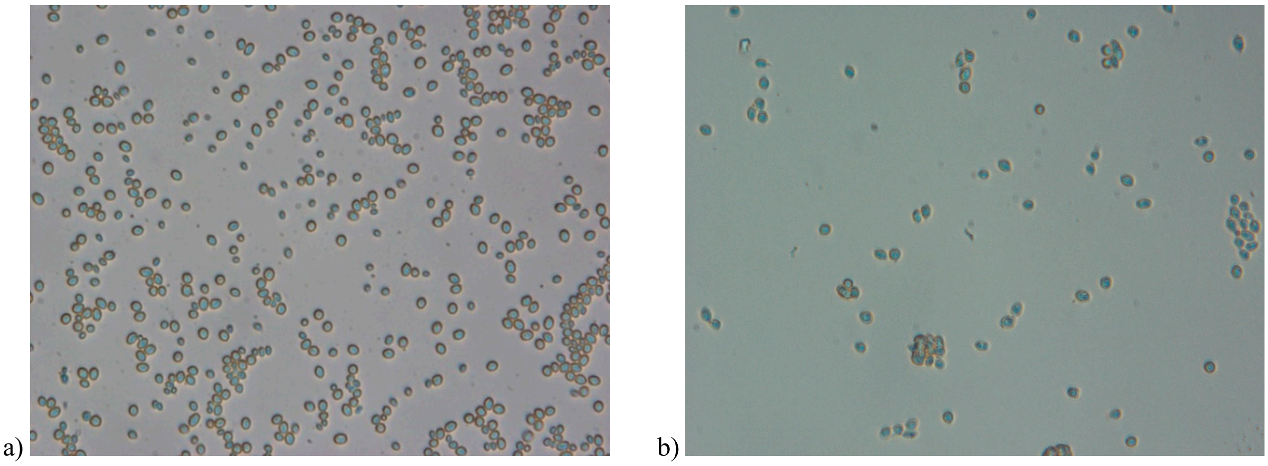

Figure S2: Observation of TT samples (magn.40×)
a) Baker's commercial yeast; b) Brewer's spent yeast

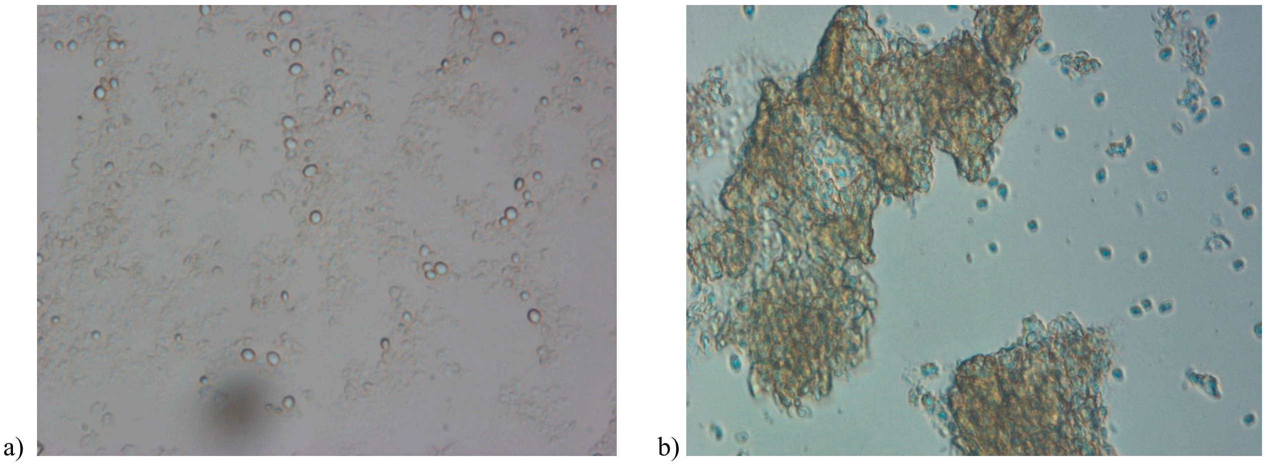

Figure S3: Observation of HPH samples (magn.40×)
a) Baker's commercial yeast; b) Brewer's spent yeast

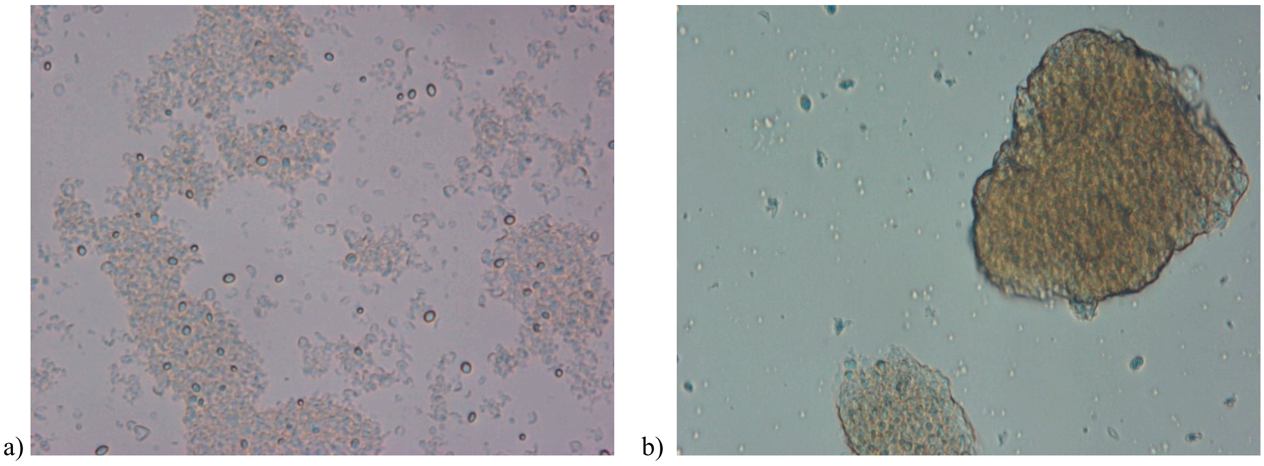

Figure S4: Observation of HPH+TT samples (magn.40×)
a) Baker's commercial yeast; b) Brewer's spent yeast

a) 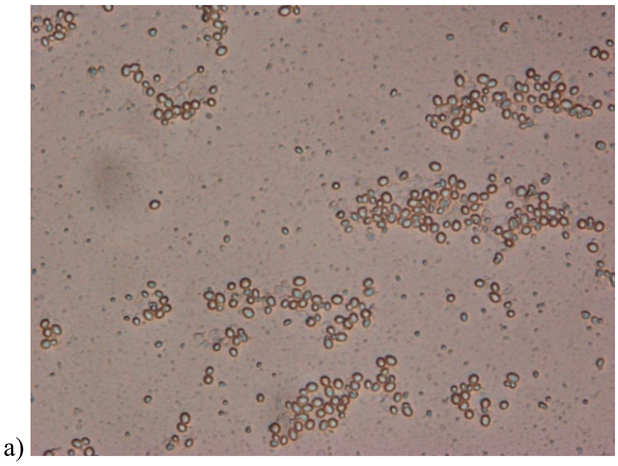

b) 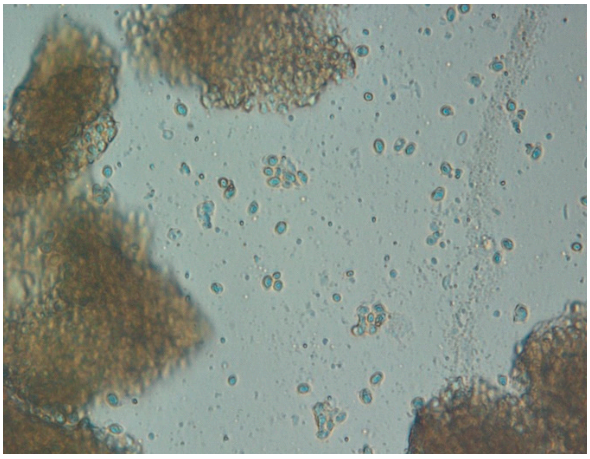

Figure S5: Observation of TT+HPH samples (magn.40×)
Baker’s commercial yeast; b) Brewer’s spent yeast

a) 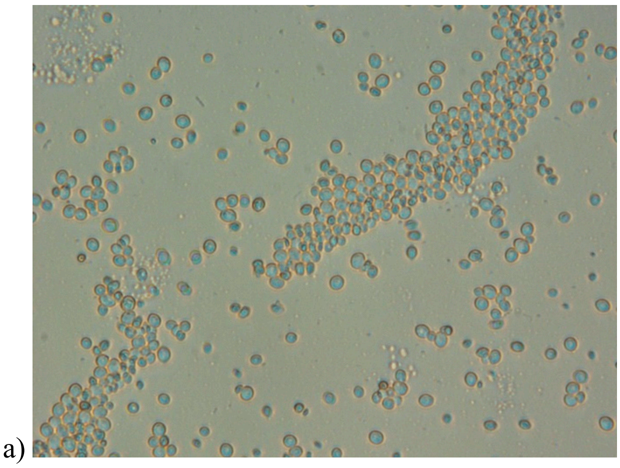

b) 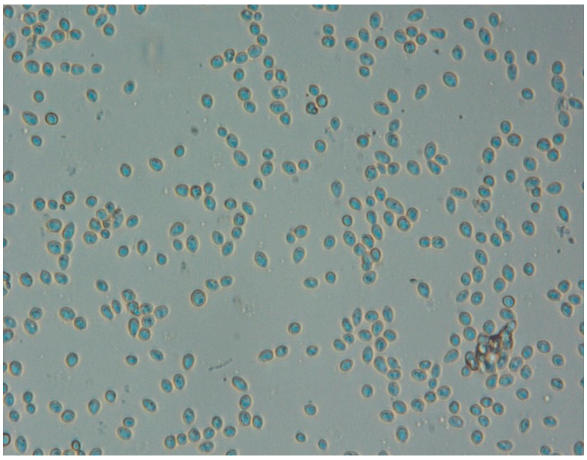

Figure S6: Observation of PEF samples (magn.40×)
a) Baker’s commercial yeast; b) Brewer’s spent yeast

a) 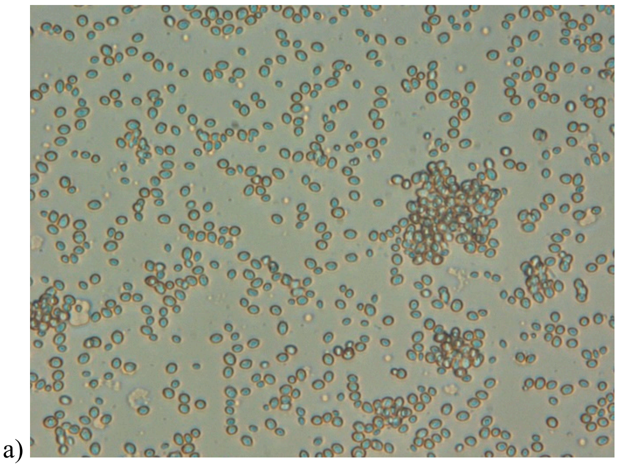

b) 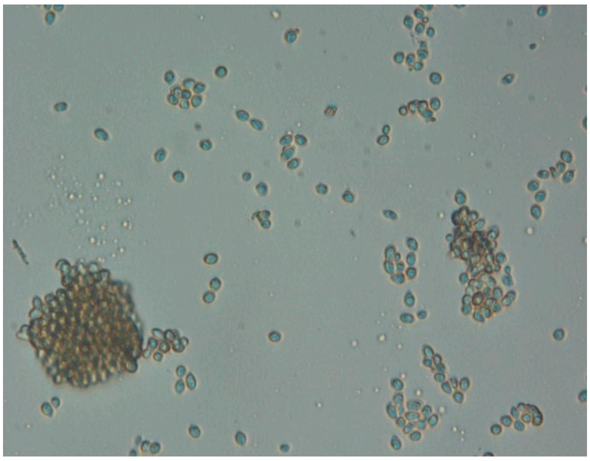

Figure S7: Observation of PEF+TT samples (magn.40×)
a) Baker’s commercial yeast; b) Brewer’s spent yeast

a) 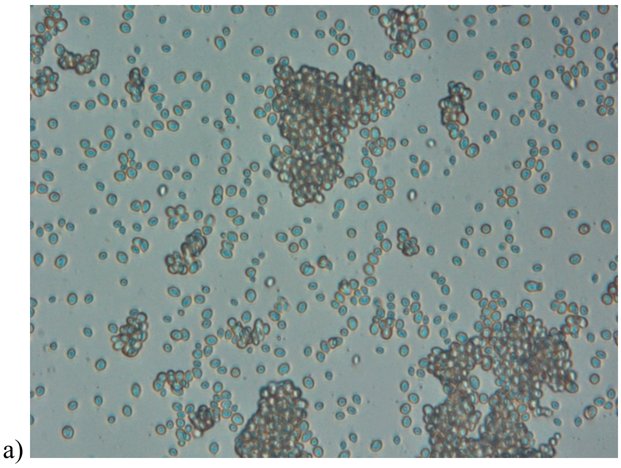 b) 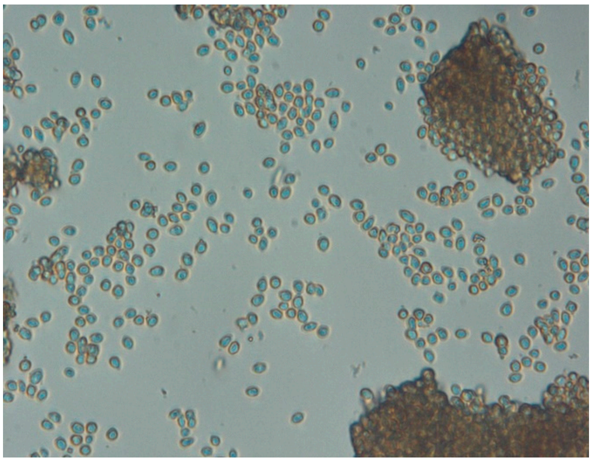

Figure S8: Observation of TT+PEF samples (magn.40×)
a) Baker’s commercial yeast; b) Brewer’s spent yeast

a) 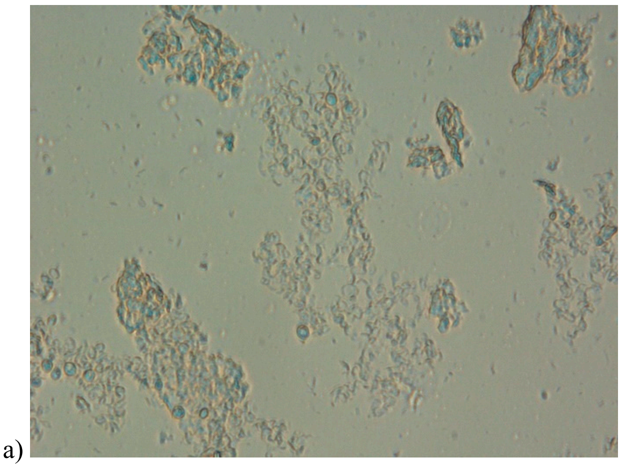 b) 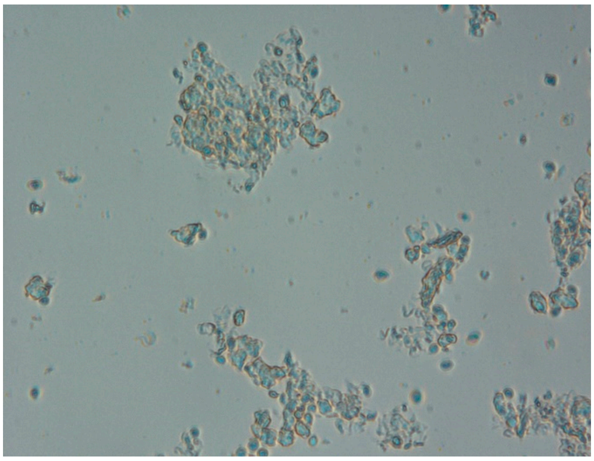

Figure S9: Observation of HPH+PEF samples (magn.40×)
a) Baker’s commercial yeast; b) Brewer’s spent yeast

a) 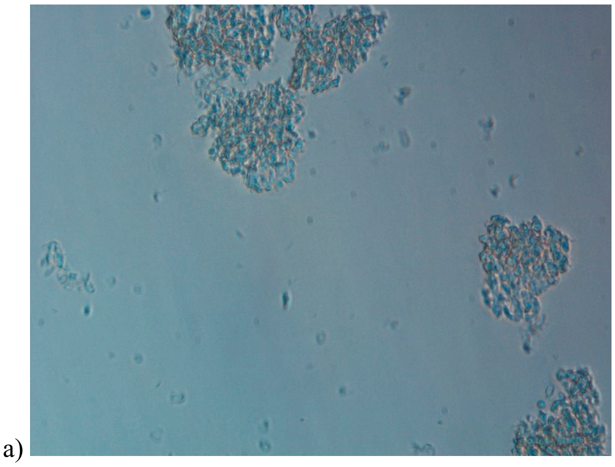 b) 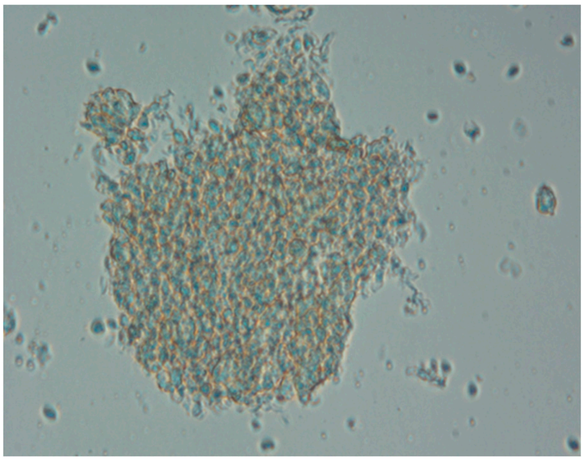

Figure S10: Observation of PEF+HPH samples (magn.40×)
a) Baker’s commercial yeast; b) Brewer’s spent yeast

***Saccharomyces cerevisiae*: Observation (magnification 100×)**

a) 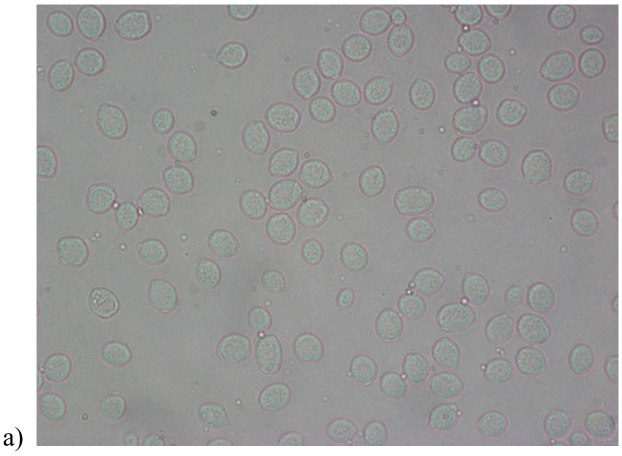 b) 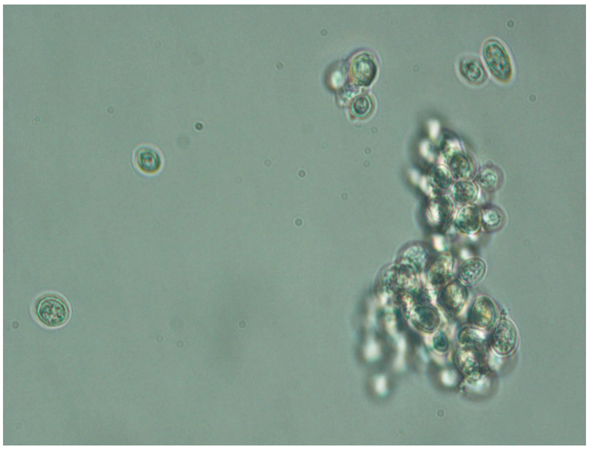

Figure S11: Observation of TT samples (magn.100×)
a) Baker's commercial yeast; b) Brewer's spent yeast

a) 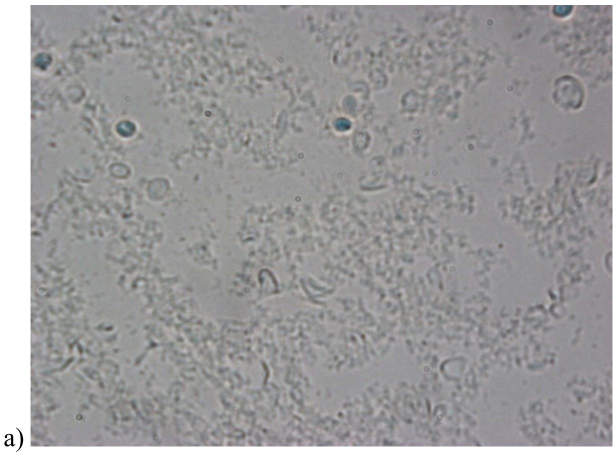 b) 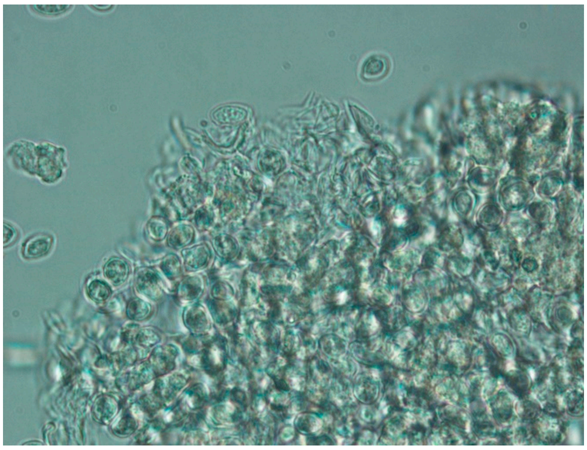

Figure S12: Observation of HPH samples (magn.100×)
a) Baker's commercial yeast; b) Brewer's spent yeast

a) 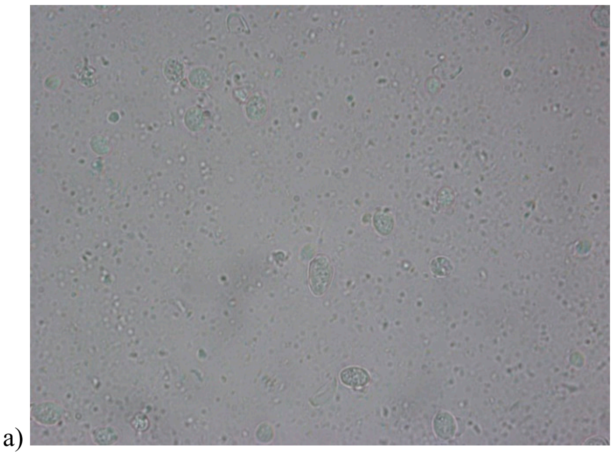 b) 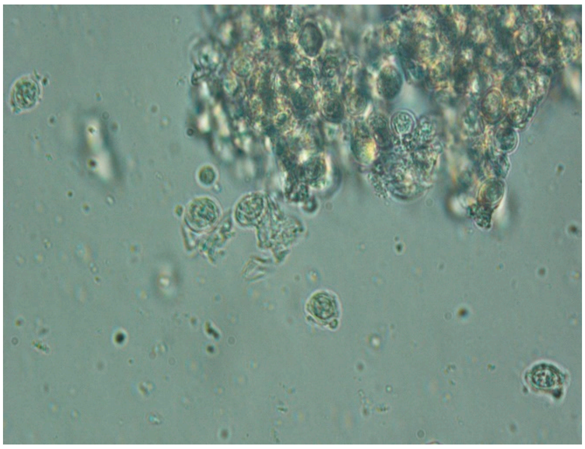

Figure S13: Observation of TT+HPH samples (magn.100×)
a) Baker's commercial yeast; b) Brewer's spent yeast

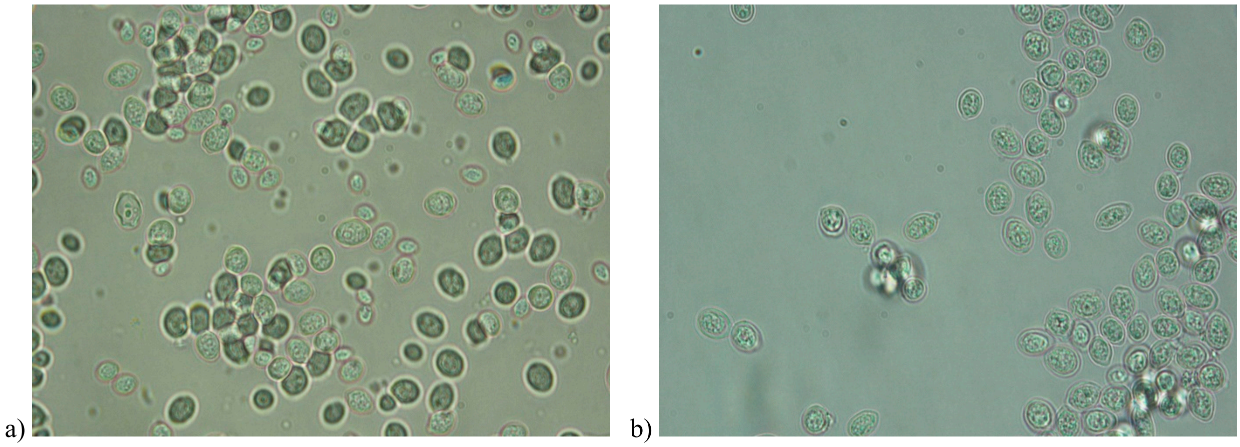

Figure S14: Observation of PEF+TT samples (magn.100×)
a) Baker's commercial yeast; b) Brewer's spent yeast

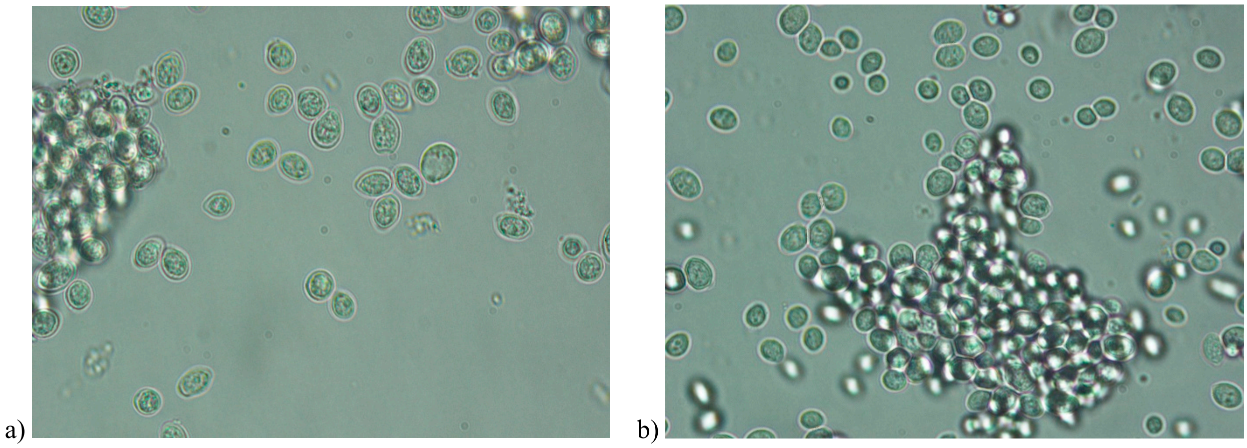

Figure S15: Observation of TT+PEF samples (magn.100×)
a) Baker's commercial yeast; b) Brewer's spent yeast

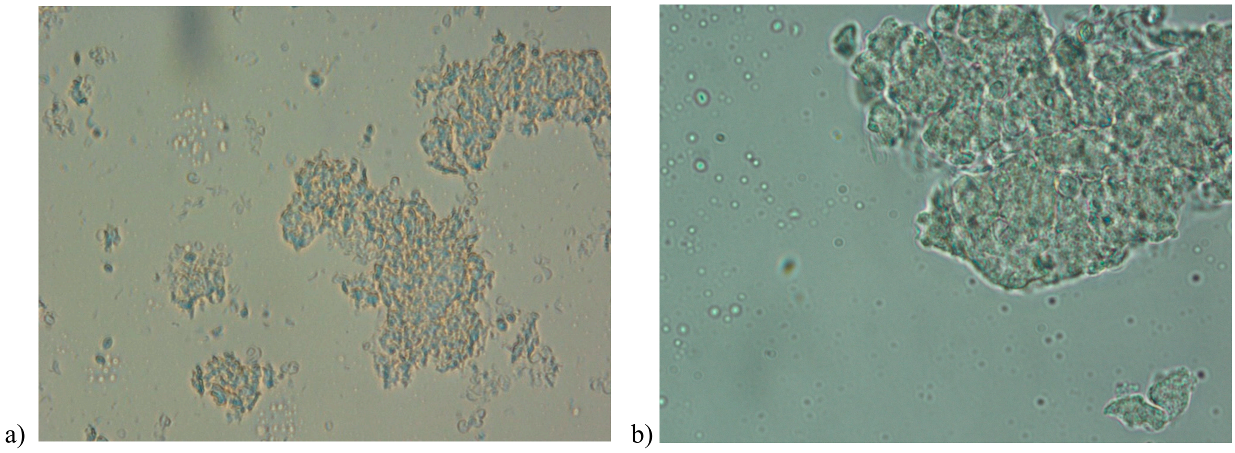

Figure S16: Observation of PEF+HPH samples (magn.100×)
a) Baker's commercial yeast; b) Brewer's spent yeast
